# Chondroitin Sulfate from *Oreochromis niloticus* Waste Reduces Leukocyte Influx in an Acute Peritonitis Model

**DOI:** 10.3390/molecules28073082

**Published:** 2023-03-30

**Authors:** Marianna Barros Silva, Lívia de Lourdes de Sousa Pinto, Luiz Henrique Medeiros, Airton Araújo Souza, Suely Ferreira Chavante, Luciana Guimarães Alves Filgueira, Rafael Barros Gomes Camara, Guilherme Lanzi Sassaki, Hugo Alexandre Oliveira Rocha, Giulianna Paiva Viana Andrade

**Affiliations:** 1 Laboratório de Biotecnologia de Polímeros Naturais (BIOPOL), Departamento de Bioquímica, Centro de Biociências, Universidade Federal do Rio Grande do Norte (UFRN), Natal 59078-970, RN, Brazil; 2 Departamento de Bioquímica, Centro de Biociências, Universidade Federal do Rio Grande do Norte (UFRN), Natal 59078-970, RN, Brazil; 3 Instituto Federal de Educação, Ciência e Tecnologia do Rio Grande do Norte, Campus de Parnamirim, Parnamirim 59143-455, RN, Brazil; 4 Departamento de Bioquímica e Biologia Molecular, Setor de Ciências Biológicas, Universidade Federal do Parana (UFPR), Curitiba 81531-980, PR, Brazil

**Keywords:** biological waste, sulfate polysaccharide, glycosaminoglycan, tilapia, inflammation

## Abstract

*Oreochromis niloticus* (tilapia) is one of the most cultivated fish species worldwide. Tilapia farming generates organic waste from fish removal processes in nurseries. Visceral waste can damage natural ecosystems. Therefore, the use of this material as a source of biomolecules helps reduce environmental impacts and improve pharmacological studies. Tilapia viscera were subjected to proteolysis and complexation with an ion-exchange resin. The obtained glycosaminoglycans were purified using ion exchange chromatography (DEAE-Sephacel). The electrophoretic profile and analysis of 1H/13C nuclear magnetic resonance (NMR) spectra allowed for the characterization of the compound as chondroitin sulfate and its sulfation position. This chondroitin was named CST. We tested the ability of CST to reduce leukocyte influx in acute peritonitis models induced by sodium thioglycolate and found a significant reduction in leukocyte migration to the peritoneal cavity, similar to the polymorphonuclear population of the three tested doses of CST. This study shows, for the first time, the potential of CST obtained from *O. niloticus* waste as an anti-inflammatory drug, thereby contributing to the expansion of the study of molecules with pharmacological functions.

## 1. Introduction

Aquaculture is increasingly recognized for its essential contribution to global food and nutrition security in the 21st century [1]. For decades, its growth rate was 8.9% per year, which corresponds to a much larger percentage than other food production sectors of animal origin. Worldwide, fish production was expected to reach approximately 178 million tons by 2020 [1,2].

Nile tilapia (*Oreochromis niloticus*) is one of the main products obtained by global aquaculture; it is estimated that 6.5 million tons are produced annually, and an average annual growth of 11% is observed in this production [3,4]. However, this cultivation method generates large amounts of waste, such as viscera discarded by producers during the fish removal process at nurseries. Tailing from this type of activity damages natural ecosystems owing to the eutrophication of streams [2]. Thus, the use of discarded waste in tilapia culture presents a significant reduction in environmental impact.

**Figure 1:**
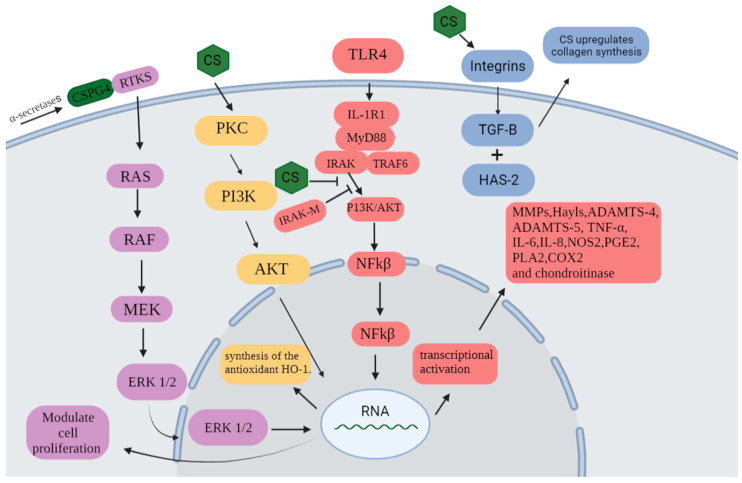
Mechanisms of action proposed for CS. Antiproliferative (purple): Proposed mechanisms of action for CS. Antiproliferative (purple): CSPG4 acts as a co-receptor to modulate RAS/MAPK signaling activity, thereby controlling RAS/MAPK cascade activity and maintaining normal proliferation levels [12]. Antioxidant (yellow): CS activates PKC, increasing PI3K and inducing synthesis of the antioxidant HO-1 [13]. Anti-inflammatory (red): CS promotes the release of IL-1 associated with IRAK-M, which decreases NFk-β translocation [10]. Treatment of osteoarthritis (blue): CS activates integrins by modulating collagen synthesis [10]. These metabolic pathways were based on Bai et al. [12], Egea et al. [13], and Bishnoi et al. [10]. CSPG4—chondroitin sulfate proteoglycan 4, RTKS—receptor tyrosine kinases, RAS-rat sarcoma GTPase, RAF—serine/threonine-specific protein kinases, MEK—mitogen-activated protein kinase, ERK ½—extracellular signal-regulated kinase 1/2, CS—chondroitin sulfate, PKC—protein kinase C, PI3K—phosphoinositide 3-kinase, AKT—protein kinase, TLR4—Toll-like receptor 4, IL-1R1—interleukin-1 receptor 1, MYD88—myeloid differentiation primary response gene 88, IRAK—interleukin-receptor-associated kinase, TRAF6—TNF-receptor-associated factor-6, IRAK-M—IRAK inhibitor, NF-κβ—nuclear factor kappa β, TGF-β—transforming growth factor beta, and HAS2—hyaluronic acid synthase-2). This figure was created with BioRender.com (17 March 2023).

During the last decades, an increasing number of bioactive compounds have been isolated from aquatic organisms, such as glycosaminoglycans (GAGs), and their therapeutic potential has been proposed [5,6], which could be a viable use for the waste. GAGs chains form part of the proteoglycans in the extracellular matrix and are responsible for much of their activity because of their potential to selectively bind many proteins and pathogens, acting in many disease processes [7]. GAGs extracted from marine animals, such as chondroitin sulfate (CS), can play a significant role in biological processes; they have antioxidant [8], antiproliferative [9], and anti-inflammatory [10] effects and play a role in the treatment of osteoarthritis [11]. Figure 1 shows some of the intracellular mechanisms of action of CS in carrying out the aforementioned activities.

Chondroitin sulfate is known to consist of disaccharide sequences alternating D-glucuronic acid (GlcA) and N-acetyl-D-galactosamine (GalNAc) linked by 𝛽-(1 → 3) glycosidic bonds [5,14]. Chondroitin sulfate has already been used in osteoarthritis (OA) treatment by reversing, retarding, or stabilizing the disease [15]. Inflammation plays an important role in the onset and progression on OA. The breakdown of cartilage that happens together with the development of the disease is related to raising inflammatory mediators [16]. In recent years, several studies have evaluated the potential of chondroitin sulfate to act in different inflammation models [17,18,19]. This study uses chondroitin sulfate obtained from tilapia viscera to evaluate its role in an acute peritoneal inflammation model in mice, thereby providing value to tilapia viscera as a raw material for obtaining chondroitin sulfate.

## 2. Results and Discussion

### 2.1. Purification and Structural Characterization of Chondroitin from O. nilotic

**Figure 2:**
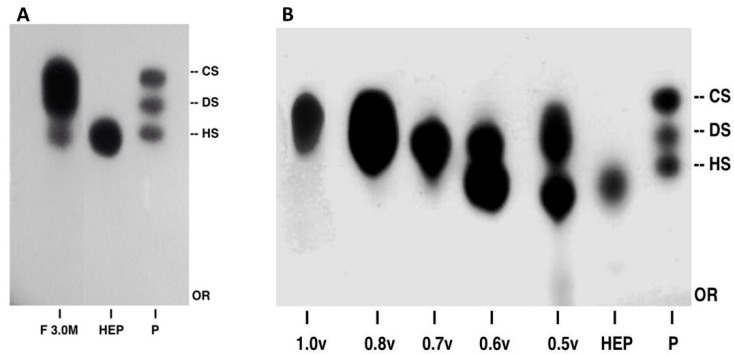
Electrophoretic migration of GAGS obtained from *O. nilotic*. (**A**) PDA electrophoresis buffer system of samples extracted after complexation. (**B**) PDA electrophoresis buffer system of samples obtained after fractionation with acetone (fractions 0.5–1.0v). P—standard of glycosaminoglycans (HS—heparan sulfate; DS—dermatan sulfate, and CS—chondroitin sulfate). HEP—heparin. Or—origin.

Acetone exposure was carried out to eliminate lipids and water, while observing that the yield in dry mass is approximately 10% of the total mass of Tilapia’s crude weight. The material was subjected to proteolysis, complexation with Lewatit’s resin, and decomplexation by elution with 3.0 M NaCl. Electrophoresis with agarose gel (Figure 2A) revealed that a pool of glycosaminoglycans was present in the fraction eluted with 3.0 M NaCl (F3.0M). The greater the interaction with diamine, the lower the electrophoretic mobility of the compound [20]. Therefore, in descending order of interactions with diamines, we have chondroitin sulfate (CS), dermatan sulfate (DS), and heparan sulphate/heparin (HS). The compounds obtained in F3.0M were represented by a metachromatic band with electrophoretic migration, similar to the glycosaminoglycans used as the standard (Figure 2A). The extraction procedure used in this study was similar to that previously described to obtain heparin-like compounds [8,21,22] because different glycosaminoglycans may be obtained with this process.

**Figure 3:**
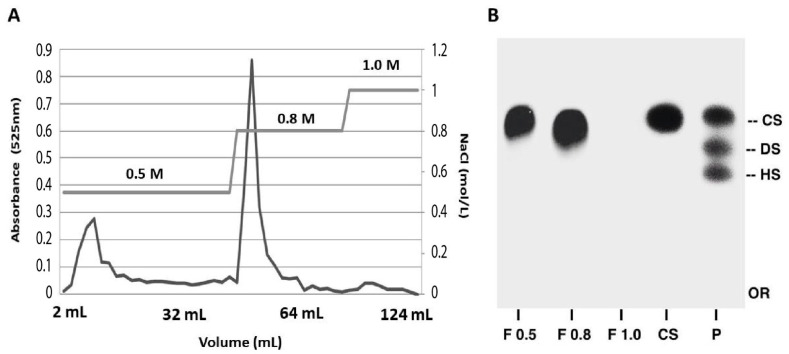
**Purification of compounds present in the fraction F0.8v using DEAE-Sephacel**. (A) The central line shows the NaCl gradient used. The fractions were monitored by measuring the absorbance at 525 nm, using uronic acid as standard. (B) PDA electrophoresis buffer system. P—standard of glycosaminoglycans (HS—heparan sulfate; DS—dermatan sulfate, and CS—chondroitin sulfate). CSA—chondroitin-4-sulfate from whale cartilage used as standard. F 0.5, F 0.8, and F 1.0—fractions of NaCl gradient used for elution. Or—origin.

Compounds present in F3.0M were fractionated with increasing proportions of acetone (0.5, 0.6, 0.7, 0.8, and 1.0v) used for the precipitation of GAGs [7]. The electrophoretic PDA profile of the compounds obtained after acetone fractionation revealed that chondroitin sulfate was present mainly in fractions F0.8v and F1.0v (Figure 2B). The presence of chondroitin sulfate in F0.8v, in addition to its higher yield than the other fractions, was considered when choosing F0.8v for purification by ion-exchange DEAE-Sephacel. Different molarities of NaCl (0.5 M, 0.8 M, and 1.0 M) were used and the eluate was monitored by uronic acid (see the Materials and Methods section). The elution profile of F0.8v is shown in Figure 2A and the two peaks that are highlighted correspond to eluted fractions with 0.5 M and 0.8 M NaCl. The isolated compounds were analyzed by electrophoresis on a PDA system (Figure 3B). The elution fractions shown in the previous figure correspond to metachromatic bands with electrophoretic migration, such as chondroitin sulfate. However, the F0.8M (prominent peak) showed 70% of the total content of uronic acid and thus was used for further studies. Considering that the compound isolated from tilapia viscera presented an electrophoretic profile similar to that of chondroitin sulfate, it was called CST.

Chondroitin sulfate has been isolated from different sources such as pig and ox trachea, sturgeon cartilage [23], chicken cartilage [24], shark cartilage [5], cartilage from different species of fish [5,25], and sub-products of mechanical chicken bones [26].

Nuclear magnetic resonance (NMR) spectroscopy is essential for structural characterization of CSA. Moreover, this technique has been reported in several studies that evaluated the structure of CSA [27]. Therefore, CST was subjected to NMR spectroscopy to confirm its identity as a chondroitin sulfate molecule. Two standard chondroitin (see the Materials and Methods section) were also subjected to NMR analysis, and their spectra were used to show the similarity between them and CST.

**Figure 4:**
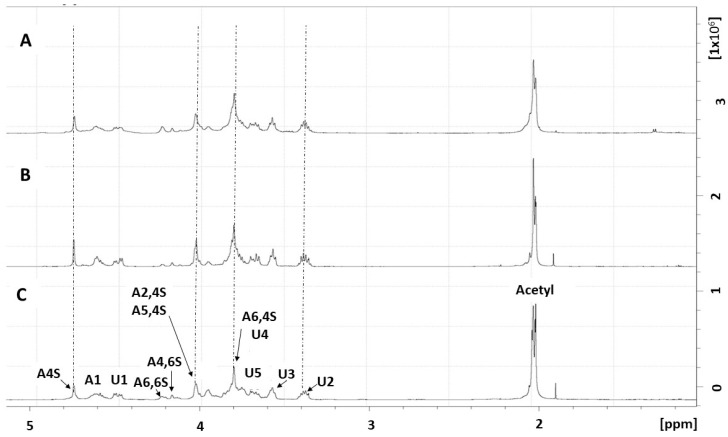
**The** 1D 1H NMR spectra of chondroitin sulfate preparations. (**A**) Tilapia; (**B**) mammalian cartilage; (**C**) marine. U, A, and S stand for glucuronic acid, N-acetylgalactosamine, and sulfate, respectively. The numbers after these letters indicate the positions of hydrogen atoms.

As shown in Figure 4, both spectra of the standard chondroitin (Figure 4B,C) show signals in the same region, demonstrating that these two molecules are structurally similar. Additionally, signals from the CST spectrum (Figure 4A) occurred in the same regions as the signals from the standard chondroitin spectra. This proves that CST is a chondroitin sulfate molecule.

The signals from the CST spectrum (Figure 4A) were then assigned based on the spectra of standard chondroitin (Figure 4B,C) and data published by other authors [28,29,30,31].

The signals at 4.49 (A1) and 4.34 (U1) ppm were assigned to the H1 of N-acetylgalactosamine and glucuronic acid, respectively. Another prominent H signal is shown in 4.73 ppm (A4S), which was assigned as H4 of 4-sulfated β-N-acetylgalactosamine (β-GalNAc4s). The prominent signal at 2.02 ppm (Figure 4A) was assigned to the acetyl group of CST. All the other proton signals of CST are found in regions of the spectrum between 3.00 and 5.00 ppm (Figure 4), as previously described for the characterization of CSA [28].

**Figure 5:**
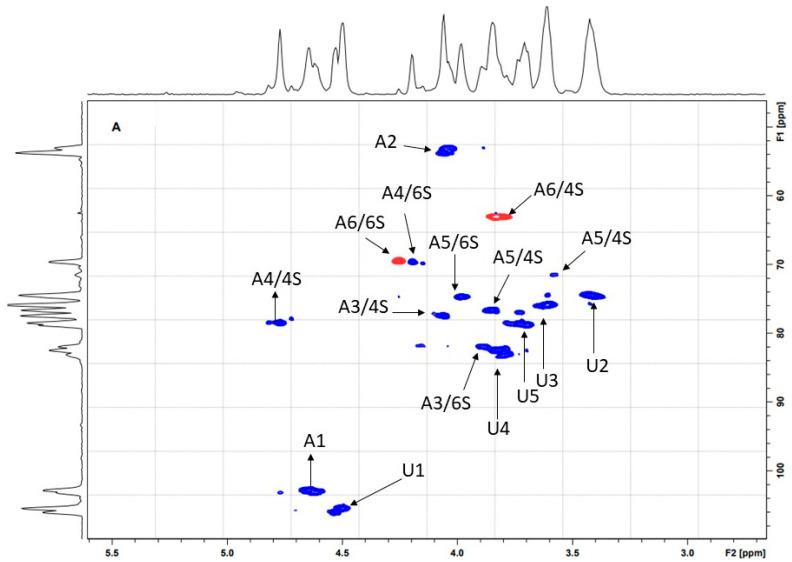
The 1H/13C NMR spectra of chondroitin sulfate isolated from the waste of *O. niloticus*. Correlation signal protons (H)/carbon (C) corresponding to the structure of chondroitin sulfate from tilapia.

The data HSQC NMR (Figure 5) confirms that the signal at 4.78 ppm represents C4 sulfation of CST. Figure 5 shows the direct correlation between the proton and carbon signals (1H/13C). The 1H/13C of CST showed signals at 3.78/64.11 ppm and 4.20/70.58 ppm representing H6/C6 of GalNAc4s and 6-sulfated β-N-acetylgalactosamine (GalNAc6S), respectively, which were identified by Maccari, Ferrarini, and Volpi [31] when they analyzed chondroitin sulfate obtained from sturgeon. In the ranges of 4.00/78.90 ppm and 3.80/77.74 ppm, the spectra refer, respectively, to the carbons H3/C3 and H5/C5 of the GalNAc4s. H2/C2 of GalNAc6S was identified at 4.01/54.65 ppm [15]. Finally, spectra were also identified in 4.49/106.98, 3.38/75.28, and 3.60/76.88 ppm which were assigned to the H1/C1, H2/C2, and H3/C3 glucuronic acid (β-GlcA), respectively. The NMR signals of theCST were identical or very close to the spectral range that describes the chondroitin obtained from vertebrate species, as described by Mucci, Schenettia, and Volpi [28].

The CSA chain may contain more than 100 disaccharide units, which can either be non-sulfated (CS-O) or sulfated at varied positions [31,32]. The sulfation pattern is an important post-translational modification that regulates the interaction of these molecules with protein chains [32,33]. The classification and type of CS depends on sulfate group placement [34]. CS heterodimers are predominantly sulfated on the hydroxyl groups of carbon 4 of galactosamine (CSA), which corresponds to the disaccharide sequence termed CSA. Disaccharides sulfated at position 6 of GalNAc are characterized as C6S, also known as CSC. Other disaccharide units undergo sulfation at more than one position, such as the disaccharides called B, D, and E. Sulfate groups linked mainly to carbon 2 of the GlcA and carbon 6 of the GalNAc characterize the disaccharide D (CSD), while the sulfation on C4 and C6 of the galactosamine determines what is known as disaccharide E (CSE) [31,35,36]. Sulfates may be present on the C2 of glucuronic acid and C4 of GalNAc (disaccharide B CS-B) [31,37]. All proton and carbon signals identified by NMR spectroscopy (Figure 4) refer to non-sulfated GlcA, GalNAc4s, or GalNAc6s. However, even if a specific type of disaccharide is predominant, disaccharides with different numbers and positions of sulfate groups can be located at different percentages within the polysaccharide chain [14,31].

The heterogeneity of the CS molecule is responsible for the variety and specificity of functions that it performs [11]. Several lines of evidence suggest that CS chains have important biological functions in inflammation [9,10], proliferation, differentiation, migration, tissue morphogenesis, organogenesis, infection, and scarring [11]. These effects are related to the capacity of chondroitin to interact with a wide variety of molecules, including matrix molecules, growth factors, protease inhibitors, cytokines, chemokines, and adhesion molecules, through specific saccharide domains within its chains [11,21].

### 2.2. Effect of Chondroitin from O. nilotic in Leukocyte Influx in Acute Peritonitis Models

CS was evaluated for its ability to modulate the inflammatory response in astrocyte cultures stimulated with lipopolysaccharide (LPS), and this study showed that the compound reduced neuroinflammation [34]. In vivo studies have shown the potential of CS to reduce the severity of arthritis in rats [14,38]. Several studies show that CS may interfere with inflammatory processes by inhibition of the expression of pro-inflammatory molecules, such as interleukin- 1 beta (IL-1β), tumor necrosis factor-alpha (TNF-α), and nuclear factor-κB (NF-kB) [39,40]. Based on these data, the isolated compound (CST) was tested in a peritoneal inflammatory model induced by sodium thioglycolate. Injection of thioglycollate into the peritoneal cavity of mice induces acute inflammation and neutrophilic infiltration [21].

Inflammation is a complex, dynamic, and well-regulated process that plays an essential role in the defense mechanisms of the body [41]. Therefore, it is vital to prevent tissue injury, which may be associated with various causes such as pathogen infection, tissue injury, malignant neoplasia, and autoimmune reactions. The inflammatory response protects the body from further damage because it signals the need to eliminate harmful factors and promote tissue repair and healing of injuries, in addition to establishing immunological memory [16,41].

**Figure 6:**
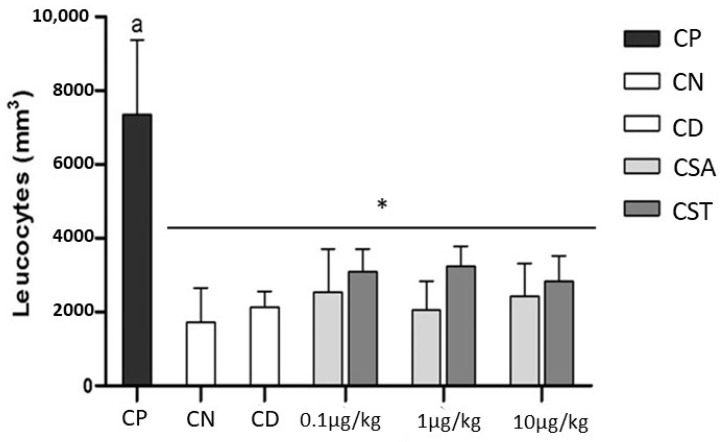
Total leukocytes count from the peritoneal fluid sample six hours after the induction of inflammation in the mice. CP—positive control. CN—negative control. DC—diclofenac (1,07 mg/kg). CSA—chondroitin-4-sulfate from whale cartilage used as standard (doses 0.10 µg/kg, 1.0 µg/kg, and 10.0 µg/kg). CST—chondroitin sulfate isolated from tilapia (doses 0.10 µg/kg, 1.0 µg/kg, and 10.0 µg/kg). The average values of leukocyte counts in peritoneal fluid that present * are statistically equal compared to the control group according to two-way ANOVA tests and Bonferroni post hoc analyses. The letter “a” above the bar indicate statistical significance compared with the CP (*p* < 0.001).

All inflammatory processes involve or depend on the recruitment of leukocytes to the sites of inflammation [42]. Since the peritoneum contains many lymphocytes, including resident peritoneal leukocytes [43], the cell number of the negative control (CN) is considered to be basal, and the difference between the positive control (CP) and CN represents the number of leukocytes that migrated after the inflammatory stimulus (100% migration). The average leukocyte count from the analysis of the peritoneal cavity of animals subjected to the experiment (Figure 6) indicated a significant increase (*p* < 0.001) in cellular influx in the CP when compared to the CN group. Therefore, in this study, the increase in leukocytes in the inflamed area of experimental animals was considered evidence of an inflammatory process.

The administration of the drug diclofenac of sodium, already known and used in human medicine [42], can reduce the migration of leukocytes by approximately 92.7%; therefore, no statistical difference exists with CN (Figure 6). However, the use of non-steroidal anti-inflammatory drugs such as diclofenac is limited because of their side effects, including gastrointestinal, renal, and hepatic lesions [42]. Thus, it is feasible to study natural molecules, such as chondroitin sulfate, which may show a similar potential.

In mice injected with CS isolated from tilapia prior to thioglycollate injection, there was a significant reduction (*p* < 0.001) in the proportions of leukocytes from the peritoneal fluid at all three tested doses compared to CP. The percentage of inhibition of leukocyte migration by CSA was 87.4% (10.0 µg/kg), 94.0% (1.0 µg/kg), and 85.8% (0.10 µg/kg). The administration of CST at a dose of 10.0 µg/kg reduced the leukocyte migration by 80.4%, whereas the dose of 1.0 µg/kg and 0.10 µg/kg of CST reduced 73.1% and 75.7% of the cellular influx, respectively. In addition, this inhibition percentage represents the potential of CST to modulate the inflammatory response in mice before the inflammatory stimulus and demonstrates that the effect of the compound does not occur in a dose-dependent manner.

The data in Figure 6 indicate that CST has similar activity to that of CSA. However, CST has an advantage in that it can be obtained from discarded material. The anti-inflammatory mechanism of action of CST has not been investigated. However, it has been demonstrated that a type of CSA inhibits specific interactions between L- and P-selectins and chemokines. These interactions are involved in leukocyte trafficking and inflammatory diseases [44]. This could be the possible mechanism of action of CST. Further studies may clarify this possibility.

**Figure 7:**
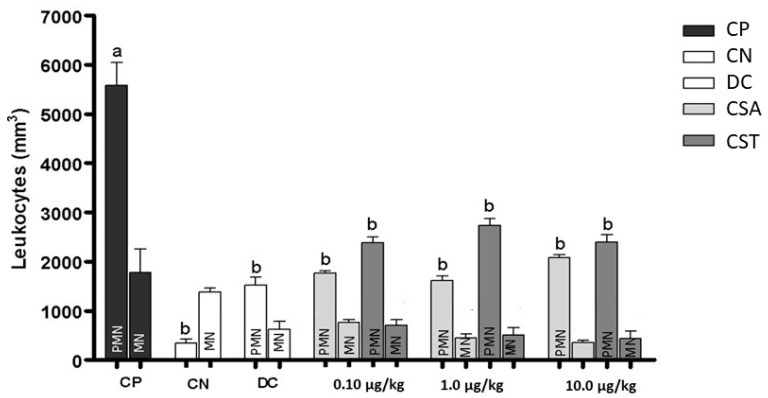
Cell differentiation in the absolute number of leukocytes polymorphonuclear and mononuclear in the peritoneal fluid six hours after the induction of inflammation in the mice. Values are expressed as mean ± standard deviation. CP—positive control. CN—negative control. DC—diclofenac (1.07 mg/kg). CSA—chondroitin sulfate standard from whale cartilage (doses 0.10 µg/kg, 1.0 µg/kg, and 10.0 µg/kg). CST—chondroitin sulfate isolated from tilapia (doses 0.10 µg/kg, 1.0 µg/kg, and 10.0 µg/kg). MN—mononuclear. PMN—polymorphonuclear. Polymorphonuclear. The letters “a” and “b” above the bars indicate statistical significance compared with the CP (*p* < 0.001).

The innate immune system responds to inflammation, infection, and injury by recruiting neutrophils and other leukocytes [45]. In this context, populations resulting from the differentiation of leukocytes after induction of inflammation were evaluated. These cells can leave the intravascular compartment through a complex process involving distinct steps that can be modulated by cytokines or chemokines [46]. Figure 7 shows that the absolute number of polymorphonuclear—PMN cells (neutrophils, eosinophils, and basophils) was higher than that of mononuclear—MN cells (lymphocytes and monocytes, in particular), except in the CN. The progression of time-dependent inflammation is triggered by specific types of leukocytes, starting with neutrophils, followed by monocytes [47].

The inflammatory response is characterized by the activation of several signaling pathways that regulate the expression of inflammatory mediators in resident tissue cells and leukocytes recruited from the blood [48,49]. Factors that trigger inflammation must be resolved to prevent the progression of other factors that promote chronic inflammation and related diseases [47]. The deregulation of the biological and molecular mechanisms involved in inflammation is related to several diseases, such as sepsis, infectious diseases, trauma, asthma, allergy, autoimmune diseases, transplant rejection, cancer, neurodegenerative diseases, obesity, and atherosclerosis [50]. However, the reduction in or removal of leukocytes from inflamed sites allows for a return to homeostasis [47].

The chondroitin sulfate obtained from biological waste and purified during this study was able to inhibit leukocyte recruitment by up to 80.4% (10.0 µg/kg). Therefore, CST represents a compound with the potential for further research aimed at fully elucidating the structure and mechanism of action in the inflammatory process and searching for new biological applications that allow for the therapeutic use of this molecule in the biotechnology field.

## 3. Materials and Methods

### 3.1. Samples/Materials

Viscera from *O. niloticus* (Linnaeus 1758) obtained from fish nurseries in Mato Grande city, state of Rio Grande do Norte, Brazil, were used as the raw material in the process of obtaining GAGs. After collection, viscera were placed in a refrigerated container and kept at −20 °C until processing. Sodium heparin from swine mucosa was obtained from Derivati Laboratory organici (Trino Vercellese, Italy). Chondroitin 4-sulfate (extracted from whale cartilage) and dermatan sulfate (extracted from bovine intestinal mucosa) were obtained from Seikagaku Kogyo Co. (Tokyo, Japan). Only analytical grade reagents were used.

### 3.2. Animals

For evaluation of leukocyte migration mice were used in the Swiss lineage, males, and females of 6–8 weeks of age and weighing 20–35g. The animals were maintained with free access to water and food in lighting-controlled conditions (12-h light/dark cycle). The survey of these animals was previously approved by the Comissão de Ética no Uso de Animais (Ethics Committee on the Use of Animals, loose translation) of Universidade Federal Rio Grande do Norte (UFRN/Brazil) where the experiments were conducted (Protocol No. 044/2010).

### 3.3. Extraction and Purification of Chondroitin Sulfate

Adult tilapia viscera were homogenized in 0.5 M NaCl solution and subjected to proteolysis by the addition of superase enzyme at the proportion of 1 g of dried mass to 20 mg of enzyme. The pH was periodically adjusted to 8.0 with the aid of sodium hydroxide (NaOH). The mixture was then covered with a layer of toluene to prevent bacterial growth and left for approximately 40 h at 60 °C. The proteolyzed mixture was filtered and Lewatit ion exchange resin (Bayer, SP, Brazil) was added to the filtrate and stirred for 24 h for the complexation of polysaccharides. The resin was collected by filtration, and the compounds complexed to the resin were eluted with increasing concentrations of NaCl. The fraction obtained with 3.0 M NaCl was then subjected to precipitation with 2 volumes of methanol, and after 18 h, the material was centrifuged at 4 °C. The resulting precipitate was vacuum-dried, solubilized in water, dialyzed, and lyophilized. The fractionation was carried out with increasing volumes of acetone (0.5 v, 0.6 v, 0.7 v, 0.8 v, and 1.0 v), and the precipitate obtained was dried and analyzed by electrophoretic behavior, as described below.

The fraction that contained the glycosaminoglycans of interest, F 0.8v, was transferred to the purification step by ion exchange chromatography DEAE-Sephacel. The sample was eluted with increasing NaCl molarities of 0.5, 0.8, and 1.0 M, respectively. The eluate was placed at refrigeration to 18 °C and subjected to monitoring of uronic acid dosage for the colorimetric reaction with carbazole, using D-glucuronic acid as standard [8].

### 3.4. Agarose Gel Electrophoresis

After each step of the process for obtaining the compounds, they were assessed by electrophoresis by the agarose gel method (0.6%) which allows for the identification of glycosaminoglycans present in the sample based on the migration profile of a GAGs mixture (heparan sulfate, dermatan sulfate, and chondroitin sulfate) used as standard [51,52]. The buffer system used was 1,3-diaminopropane acetate (PDA) 0.05M; pH 9.0. aliquots (5 μg) of the fractions were applied to the gel and subjected to electrophoresis. The gels were fixed with 0.1% cetyltrimethylammonium bromide solution (CETAVLON), dried under hot air stream, stained with blue toluidine (0.1% acetic acid and 50% ethanol), and further bleached with the same solution without the dye [20].

### 3.5. NMR Spectroscopy

All chondroitin sulfate preparations (CST, CS marine EDQM (Y0000593) and CS leaflet EDQM (Y0000280) had their pHs adjusted to 7.0. Ten mg of each polysaccharide was dissolved in 0.5 mL of D_2_O and chemical shifts were referenced with TMSP-d4 (2,2,3,3-tetradeuterium-3-trimethylilsilylpropionate, δ = 0). Structural characterization was performed by NMR using a Bruker spectrometer AVANCE III 600 MHz TCI 5 mm N2 Prodigy equipped with a 14.1 Tesla magnet. One-dimensional and two-dimensional NMR chemical shifts of ^1^H and ^13^C were determined at 343 K. Two-dimensional edited HSQC experiments were recorded for quadrature detection in the indirect dimension and acquired using eight scans per series of 2048 × 320 data points, with no zero filling in F1 (1024) prior to Fourier transformation.

### 3.6. Effect of Chondroitin Sulfate from Tilapia on Inflammation

The anti-inflammatory potential of chondroitin sulfate was evaluated through the sodium-thioglycollate-induced peritonitis model [52]. Groups of 06 (six) mice were weighed and identified on the day before the experiment, and injectable solutions were prepared under sterile conditions (laminar flow hood), as will be detailed. In tests with control groups, the mice did not receive any treatment because both groups received only subcutaneous injection of sterile saline (200 μL). After 30 min, 1 mL of intraperitoneally saline solution (negative control) or 1 mL of 3% thioglycollate (positive stimulus for inflammation of the peritoneum) was administered to all animals except the negative control ones.

Assays with the GAGs were developed in a similar way, considering that the animals were pretreated with the standard glycosaminoglycan, the chondroitin-4-sulfate (Sigma), or chondroitin sulfate obtained from tilapia, both diluted in 200 μL sterile saline solution at doses of 0.10 µg/kg, 1.0 µg/kg, and 10.0 µg/kg. Sodium diclofenac (Voltaren^®^), an anti-inflammatory widely used in clinical practice, was also prepared under sterile conditions applied as pretreatment of mice at a dose of 1.07 mg/kg. Subsequently, the process of inflammation was induced in all animals over a period of six (06) hours. The effect of the compound was evaluated based on the total count and differential of cell populations that were stimulated to migrate to the peritoneum.

After inducing peritoneal inflammation for six hours, a lethal dose of sodium thiopental (Thiopentax^®^) at a concentration of 20 mg/mL was administered to each animal. The animals were still alive, but under the effect of the anesthetic. Then, the abdominal cavity of the mice was washed with 4 mL of saline solution for 30 s, and 3 mL peritoneal fluid (PF) was collected from each animal. PF was placed in tubes with EDTA (BD Vacutainer^®^), and then transferred to falcon tubes and centrifuged at 405× *g* for 3 min at 4 °C. The supernatant was discarded, and the cell pellet resuspended in 500 μL of saline solution. Total leukocyte counting was carried out using a Neubauer chamber in 40x objective after diluting 20 μL of each sample in 380 μL (1:20 dilution) of Turk’s solution (3% acetic acid) under light microscopy.

From the same cell suspension, aliquots were taken to prepare slides for differential counting of leukocyte populations in the peritoneal fluid. Smears were prepared in duplicate using a specific centrifuge (Cytospin) device that performs centrifugation of the sample (137.5× *g*, for 4 min at room temperature) and concentrates cells in a central area of the blade. The slides were stained with Panopticon (LB, LaborClin^®^), and different cell populations were counted using a light microscope and a 100X lens with the aid of a blood cells manual meter. The absolute number of polymorphnuclear (PMN) and mononuclear (MN) cells in the peritoneal cavity of the studied animals was evaluated.

### 3.7. Statistical Analysis

Statistical analyses were performed using GraphPad Prism (v.6.01) (CA, USA). The results are represented as the mean ± standard error of the mean (SEM) of three individual experiments. Analysis of variance (ANOVA) followed by Tukey’s test was used to compare the means between the analyzed groups; statistical significance was established at *p* < 0.05.

## 4. Conclusions

In this study, researchers extracted and characterized glycosaminoglycan from tilapia viscera. The identity of the glycosaminoglycan was confirmed through physicochemical and NMR analyses. In vivo experiments showed that chondroitin from tilapia (CST), when administered, significantly reduced the influx of polymorphonuclear (PMN) leukocytes into the peritoneal cavity of animals compared to a control group at all evaluated doses. This suggests that CST is a potential anti-inflammatory agent. However, the mechanism of action of CST was not elucidated in this study, and future experiments are planned in order to investigate this further, as well as to investigate whether CST has other mechanisms of anti-inflammatory action, such as those described for chondroitins from other sources (Figure 1).

The structure of chondroitins from different sources can change with animal age [52], and it is unknown if this also occurs in tilapia. The correlation between CST structure and activity will be investigated in future studies.

## Data Availability

Not applicable.

## Sample Availability

Samples of the compounds are available from the authors.

## References

[1] FAO (2022). The State of World Fisheries and Aquaculture 2022.

[2] Naylor R.L., Hardy R.W., Buschmann A.H., Bush S.R., Cao L., Klinger D.H., Little D.C., Lubchenco J., Shumway S.E., Troell M. (2021). A 20-Year Retrospective Review of Global Aquaculture. Nature.

[3] FAO (2020). The State of World Fisheries and Aquaculture 2020.

[4] Gallo N., Natali M.L., Quarta A., Gaballo A., Terzi A., Sibillano T., Giannini C., De Benedetto G.E., Lunetti P., Capobianco L. (2022). Aquaponics-Derived Tilapia Skin Collagen for Biomaterials Development. Polymers.

[5] Abdallah M.M., Fernández N., Matias A.A., do Rosário Bronze M. (2020). Hyaluronic Acid and Chondroitin Sulfate from Marine and Terrestrial Sources: Extraction and Purification Methods. Carbohydr. Polym..

[6] Laurienzo P. (2010). Marine Polysaccharides in Pharmaceutical Applications: An Overview. Mar. Drugs.

[7] Mohamed S., Coombe D. (2017). Heparin Mimetics: Their Therapeutic Potential. Pharmaceuticals.

[8] Medeiros L.H.C., Vasconcelos B.M.F., Silva M.B., Souza-Junior A.A., Chavante S.F., Andrade G.P.V. (2021). Chondroitin Sulfate from Fish Waste Exhibits Strong Intracellular Antioxidant Potential. Braz. J. Med. Biol. Res..

[9] Francos-Quijorna I., Sánchez-Petidier M., Burnside E.R., Badea S.R., Torres-Espin A., Marshall L., de Winter F., Verhaagen J., Moreno-Manzano V., Bradbury E.J. (2022). Chondroitin Sulfate Proteoglycans Prevent Immune Cell Phenotypic Conversion and Inflammation Resolution via TLR4 in Rodent Models of Spinal Cord Injury. Nat. Commun..

[10] Bishnoi M., Jain A., Hurkat P., Jain S.K. (2016). Chondroitin Sulphate: A Focus on Osteoarthritis. Glycoconj. J..

[11] Habuchi O. (2022). Functions of Chondroitin/Dermatan Sulfate Containing GalNAc4,6-Disulfate. Glycobiology.

[12] Bai Z., Qu Y., Shi L., Li X., Yang Z., Ji M., Hou P. (2021). Identification of a Germline CSPG4 Variation in a Family with Neurofibromatosis Type 1-like Phenotype. Cell Death Dis..

[13] Egea J., García A.G., Verges J., Montell E., López M.G. (2010). Antioxidant, Antiinflammatory and Neuroprotective Actions of Chondroitin Sulfate and Proteoglycans. Osteoarthr. Cartil..

[14] Bauerova K., Ponist S., Kuncirova V., Mihalova D., Paulovicova E., Volpi N. (2011). Chondroitin Sulfate Effect on Induced Arthritis in Rats. Osteoarthr. Cartil..

[15] Volpi N. (2006). Therapeutic Applications of Glycosaminoglycans. CMC.

[16] Pecchi E., Priam S., Mladenovic Z., Gosset M., Saurel A.-S., Aguilar L., Berenbaum F., Jacques C. (2012). A Potential Role of Chondroitin Sulfate on Bone in Osteoarthritis: Inhibition of Prostaglandin E2 and Matrix Metalloproteinases Synthesis in Interleukin-1β- Stimulated Osteoblasts. Osteoarthr. Cartil..

[17] Chen S., Chen W., Chen Y., Mo X., Fan C. (2021). Chondroitin Sulfate Modified 3D Porous Electrospun Nanofiber Scaffolds Promote Cartilage Regeneration. Mater. Sci. Eng. C.

[18] Guan T., Ding L.-G., Lu B.-Y., Guo J.-Y., Wu M.-Y., Tan Z.-Q., Hou S.-Z. (2022). Combined Administration of Curcumin and Chondroitin Sulfate Alleviates Cartilage Injury and Inflammation via NF-ΚB Pathway in Knee Osteoarthritis Rats. Front. Pharmacol..

[19] Stellavato A., Restaino O.F., Vassallo V., Cassese E., Finamore R., Ruosi C., Schiraldi C. (2021). Chondroitin Sulfate in USA Dietary Supplements in Comparison to Pharma Grade Products: Analytical Fingerprint and Potential Anti-Inflammatory Effect on Human Osteoartritic Chondrocytes and Synoviocytes. Pharmaceutics.

[20] Presa F.B., Marques M.L.M., Viana R.L.S., Nobre L.T.D.B., Costa L.S., Rocha H.A.O. (2018). The protective role of sulfated poly-saccharides from green seaweed Udotea flabellum in cells exposed to oxidative damage. Mar. Drugs.

[21] Brito A.S., Cavalcante R.S., Palhares L.C.G.F., Hughes A.J., Andrade G.P.V., Yates E.A., Nader H.B., Lima M.A., Chavante S.F. (2014). A Non-Hemorrhagic Hybrid Heparin/Heparan Sulfate with Anticoagulant Potential. Carbohydr. Polym..

[22] Chavante S.F., Santos E.A., Oliveira F.W., Guerrini M., Torri G., Casu B., Dietrich C.P., Nader H.B. (2000). A Novel Heparan Sulphate with High Degree of N-Sulphation and High Heparin Cofactor-II Activity from the Brine Shrimp Artemia Franciscana. Int. J. Biol. Macromol..

[23] Maccari F., Ferrarini F., Volpi N. (2010). Structural Characterization of Chondroitin Sulfate from Sturgeon Bone. Carbohydr. Res..

[24] Wang X., Shen Q., Zhang C., Jia W., Han L., Yu Q. (2019). Chicken Leg Bone as a Source of Chondroitin Sulfate. Carbohydr. Polym..

[25] Lu J., Ai C., Guo L., Fu Y., Cao C., Song S. (2017). Characteristic Oligosaccharides Released from Acid Hydrolysis for the Structural Analysis of Chondroitin Sulfate. Carbohydr. Res..

[26] Nakano T., Pietrasik Z., Ozimek L., Betti M. (2012). Extraction, Isolation and Analysis of Chondroitin Sulfate from Broiler Chicken Biomass. Process Biochem..

[27] Song Y., Zhang F., Linhardt R.J. (2021). Analysis of the Glycosaminoglycan Chains of Proteoglycans. J. Histochem. Cytochem..

[28] Mucci A. (2000). 1H and 13C Nuclear Magnetic Resonance Identification and Characterization of Components of Chondroitin Sulfates of Various Origin. Carbohydr. Polym..

[29] Di Muzio L., Paolicelli P., Trilli J., Petralito S., Carriero V.C., Brandelli C., Spano M., Sobolev A.P., Mannina L., Casadei M.A. (2022). Insights into the Reaction of Chondroitin Sulfate with Glycidyl Methacrylate: 1D and 2D NMR Investigation. Carbohydr. Polym..

[30] Mourão P.A.S., Pereira M.S., Pavão M.S.G., Mulloy B., Tollefsen D.M., Mowinckel M.-C., Abildgaard U. (1996). Structure and Anticoagulant Activity of a Fucosylated Chondroitin Sulfate from Echinoderm. J. Biol. Chem..

[31] Volpi N. (2010). Quality of Different Chondroitin Sulfate Preparations in Relation to Their Therapeutic Activity. J. Pharm. Pharmacol..

[32] Flangea C., Serb A., Schiopu C., Tudor S., Sisu E., Seidler D., Zamfir A. (2009). Discrimination of GalNAc (4S/6S) Sulfation Sites in Chondroitin Sulfate Disaccharides by Chip-Based Nanoelectrospray Multistage Mass Spectrometry. Open Chem..

[33] Santos G.R., Porto A.C., Soares P.A., Vilanova E., Mourão P.A. (2017). Exploring the Structure of Fucosylated Chondroitin Sulfate through Bottom-up Nuclear Magnetic Resonance and Electrospray Ionization-High-Resolution Mass Spectrometry Approaches. Glycobiology.

[34] Cañas N., Gorina R., Planas A.M., Vergés J., Montell E., García A.G., López M.G. (2010). Chondroitin Sulfate Inhibits Lipopolysaccharide-Induced Inflammation in Rat Astrocytes by Preventing Nuclear Factor Kappa B Activation. Neuroscience.

[35] Krylov V.B., Grachev A.A., Ustyuzhanina N.E., Ushakova N.A., Preobrazhenskaya M.E., Kozlova N.I., Portsel M.N., Konovalova I.N., Novikov V.Y., Siebert H.-C. (2011). Preliminary Structural Characterization, Anti-Inflammatory and Anticoagulant Activities of Chondroitin Sulfates from Marine Fish Cartilage. Russ. Chem. Bull..

[36] Crespo D., Asher R.A., Lin R., Rhodes K.E., Fawcett J.W. (2007). How Does Chondroitinase Promote Functional Recovery in the Damaged CNS?. Exp. Neurol..

[37] Yamada S., Sugahara K. (2008). Potential Therapeutic Application of Chondroitin Sulfate/Dermatan Sulfate. CDDT.

[38] Sharma S., Lee A., Choi K., Kim K., Youn I., Trippel S.B., Panitch A. (2013). Biomimetic Aggrecan Reduces Cartilage Extracellular Matrix from Degradation and Lowers Catabolic Activity in Ex Vivo and in Vivo Models. Macromol. Biosci..

[39] Luan J., Peng X., Lin J., Zhang Y., Tian X., Zhan L., Zhao G. (2022). The Therapeutic Potential of Chondroitin Sulfate in Aspergillus Fumigatus Keratitis. Mol. Immunol..

[40] Singh S., Singh T.G., Singh M., Najda A., Nurzyńska-Wierdak R., Almeer R., Kamel M., Abdel-Daim M.M. (2021). Anticonvulsive Effects of Chondroitin Sulfate on Pilocarpine and Pentylenetetrazole Induced Epileptogenesis in Mice. Molecules.

[41] Yeung Y.T., Aziz F., Guerrero-Castilla A., Arguelles S. (2018). Signaling Pathways in Inflammation and Anti-Inflammatory Therapies. CPD.

[42] Barros-Gomes J.A.C., Nascimento D.L.A., Silveira A.C.R., Silva R.K., Gomes D.L., Melo K.R.T., Almeida-Lima J., Camara R.B.G., Silva N.B., Rocha H.A.O. (2018). In Vivo Evaluation of the Antioxidant Activity and Protective Action of the Seaweed Gracilaria Birdiae. Oxid. Med. Cell. Longev..

[43] Kipari T., Watson S., Houlberg K., Lepage S., Hughes J., Cailhier J.-F. (2009). Lymphocytes Modulate Peritoneal Leukocyte Recruitment in Peritonitis. Inflamm. Res..

[44] Sugahara K., Mikami T., Uyama T., Mizuguchi S., Nomura K., Kitagawa H. (2003). Recent Advances in the Structural Biology of Chondroitin Sulfate and Dermatan Sulfate. Curr. Opin. Struct. Biol..

[45] Schmidt S., Moser M., Sperandio M. (2013). The Molecular Basis of Leukocyte Recruitment and Its Deficiencies. Mol. Immunol..

[46] Parish C.R. (2006). The Role of Heparan Sulphate in Inflammation. Nat. Rev. Immunol..

[47] Serhan C.N., Chiang N., Van Dyke T.E. (2008). Resolving Inflammation: Dual Anti-Inflammatory and pro-Resolution Lipid Mediators. Nat. Rev. Immunol..

[48] Lawrence T. (2009). The Nuclear Factor NF- B Pathway in Inflammation. Cold Spring Harb. Perspect. Biol..

[49] Geering B., Stoeckle C., Conus S., Simon H.-U. (2013). Living and Dying for Inflammation: Neutrophils, Eosinophils, Basophils. Trends Immunol..

[50] Vodovotz Y., Constantine G., Faeder J., Mi Q., Rubin J., Bartels J., Sarkar J., Squires R.H., Okonkwo D.O., Gerlach J. (2010). Translational Systems Approaches to the Biology of Inflammation and Healing. Immunopharmacol. Immunotoxicol..

[51] Jaques L.B., Balueux R.E., Dietrich C.P., Kavanagh L.W. (1968). A Microelectrophoresis Method for Heparin. Can. J. Physiol. Pharmacol..

[52] Xie X., Rivier A.-S., Zakrzewicz A., Bernimoulin M., Zeng X.-L., Wessel H.P., Schapira M., Spertini O. (2000). Inhibition of Selectin-Mediated Cell Adhesion and Prevention of Acute Inflammation by Nonanticoagulant Sulfated Saccharides. J. Biol. Chem..

